# DIFFERENT APPROACH TO BONE MINERAL DENSITY IN NON-TRAUMATIC SPINAL CORD INJURY: A COMPARISON WITH TRAUMATIC SPINAL CORD INJURY

**DOI:** 10.2340/jrm.v58.44836

**Published:** 2026-03-17

**Authors:** Onyoo KIM, Jisun LIM, Geun-Young PARK

**Affiliations:** 1 Department of Rehabilitation Medicine, National Rehabilitation Center, Seoul; 2 Department of Medicine, Graduate School, The Catholic University of Korea, Seoul; 3 Department of Clinical Research on Rehabilitation, National Rehabilitation Center, Seoul; 4 Department of Rehabilitation Medicine, College of Medicine, The Catholic University of Korea, Seoul, South Korea

**Keywords:** bone mineral density, non-traumatic spinal cord injury, spinal cord injury, osteoporosis

## Abstract

**Objective:**

The incidence of non-traumatic spinal cord injury (NTSCI) is rising with an ageing population. This study identified diagnostic bone sites and risk factors for low bone mineral density (BMD) in patients with NTSCI and those with TSCI according to age category.

**Design:**

Retrospective cross-sectional study.

**Subjects/Patients:**

1,159 with TSCI and 475 with NTSCI at the National Rehabilitation Center in Korea.

**Methods:**

Diagnostic bone sites and BMD using dual-energy X-ray absorptiometry (DXA) were compared between groups and BMD risk factors were assessed.

**Results:**

The lumbar spine BMD and T-score values of osteoporosis were lower in the NTSCI group than in the TSCI group over 50 years old (*p* < 0.001, *p* < 0.001). There was no significant difference in the DXA results between the NTSCI and TSCI under 50 years of age. As an osteoporotic diagnostic site in the NTSCI group (≥ 50 years) (43%), the lumbar spine had higher proportions than in the TSCI group (29%). Female sex and low body mass index were risk factors for osteoporosis in both groups (≥ 50 years).

**Conclusion:**

BMD evaluation in patients aged 50 years and older with NTSCI should include the lumbar spine area. Individuals with risk factors for SCI must undergo BMD assessment and close monitoring.

## LAY ABSTRACT

The incidence of non-traumatic spinal cord injury (NTSCI) is rising with an ageing population. This study identified diagnostic bone sites and risk factors for low bone mineral density (BMD) in patients with NTSCI and TSCI categorized by age. Some 1,159 and 475 patients had TSCI and NTSCI, respectively. BMD, Z-scores and T-scores were determined using dual-energy X-ray absorptiometry. In individuals over 50 years old, the lumbar spine BMD and T-score values for osteoporosis were lower in the NTSCI group than in the TSCI group. There was no significant difference in the DXA results between the NTSCI and the TSCI groups under 50 years old. As an osteoporotic diagnostic site in the NTSCI group (≥ 50 years) (43%), the lumbar spine had higher proportions than in the TSCI group (29%). Female sex and low body mass index were risk factors for osteoporosis in both groups (≥ 50 years).

An imbalance between increased bone resorption and diminished bone formation causes a loss of bone mineral density (BMD) after spinal cord injury (SCI) (1, 2). Joint unloading, neural lesions, and hormonal changes following SCI are known contributors to osteoporosis development (3). The lack of vertical loading and paralysis of muscles on sub-lesional bone sites result in rapid and extensive bone loss and fractures (3–6). Moreover, increased osteoporosis and fractures following SCI are associated with morbidity, mortality, and substantial costs to the healthcare system (6).

In a healthy population, the International Society for Clinical Densitometry recommends the dual-energy X-ray absorptiometry (DXA) test for all women aged ≥ 65 years and in men aged > 70 years (7). Compared with non-disabled individuals, SCI is a risk factor for bone loss (7). Risk factors for low bone mass following SCI include age, sex, level of injury, severity of traumatic SCI (TSCI), and injury duration (8–11). Furthermore, the most common fracture sites after SCI are the hip, distal femur, and proximal tibia (12).

Numerous BMD studies on TSCI have been published over many years (6, 8). However, the incidence of non-traumatic SCI (NTSCI) has increased steadily due to the rapid rise in the older population (13). Studies on BMD in patients with NTSCI have also been conducted rarely, and evidence of differences in BMD between NTSCI and TSCI has not been investigated. Compared with individuals with TSCI, those with NTSCI exhibit different characteristics in terms of sex predominance, mean age, and injury level and severity (14). Further studies on BMD loss in individuals with NTSCI are needed to identify diagnostic bone sites and risk factors for osteoporosis.

This study is the first trial to determine the characteristics of osteoporosis in patients with NTSCI and compare them with those of patients with TSCI according to age category. The main hypotheses were as follows: (*i*) BMD in individuals with NTSCI will have different characteristics, including diagnostic bone sites, compared with those in individuals with TSCI; (*ii*) NTSCI and TSCI have different risk factors for BMD based on DXA testing.

## METHODS

### Participants

**Fig. 1:**
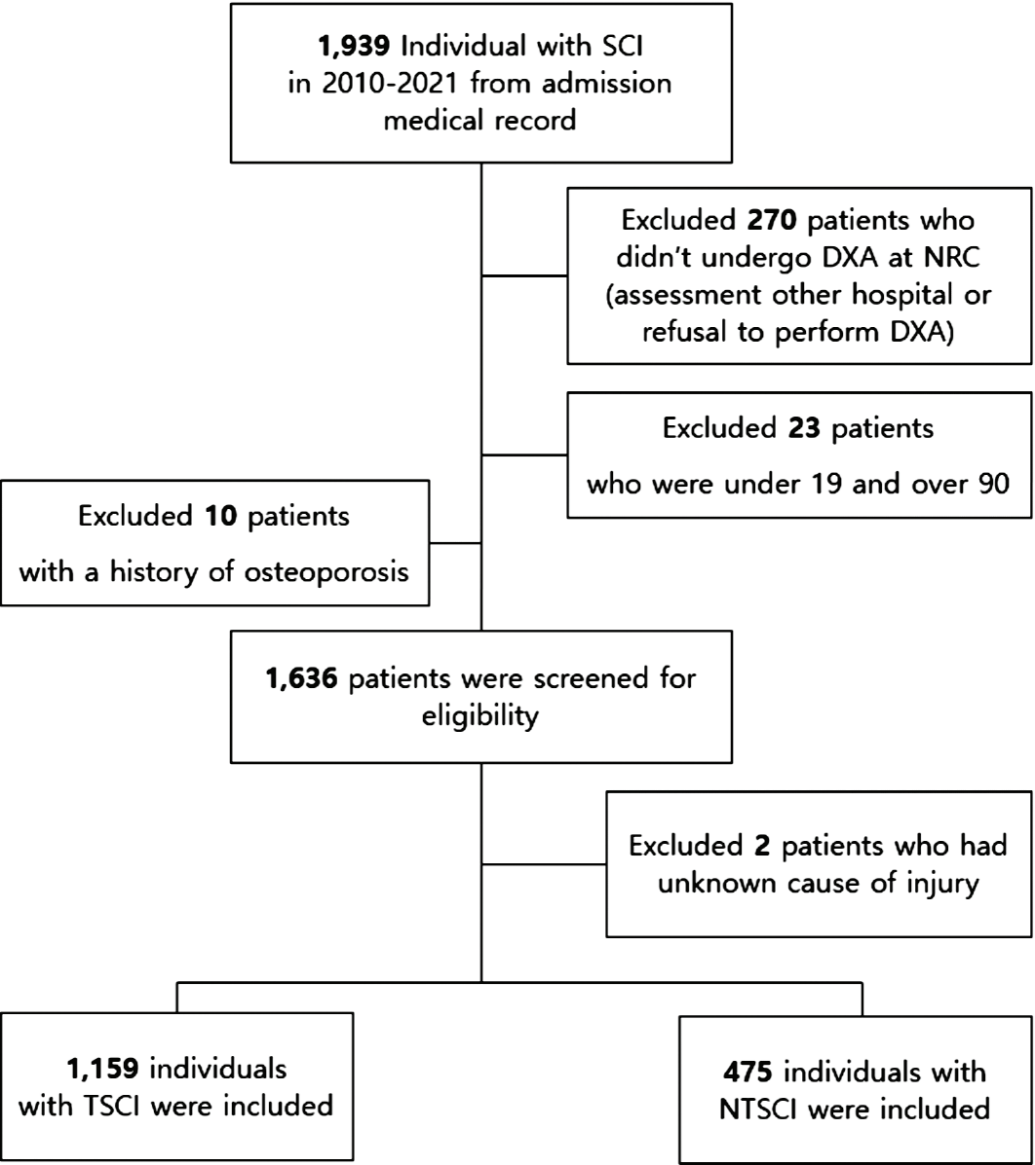
Participant flowchart. SCI: spinal cord injury; DXA: dual-energy X-ray absorptiometry; NTSCI: non-traumatic spinal cord injury; TSCI: traumatic spinal cord injury; NRC: national rehabilitation centre.

Data were collected through a medical chart review of inpatients with SCI admitted to the SCI unit at a rehabilitation centre in Korea between January 2010 and October 2021. Among the initial 1,939 individuals reviewed, 270 who did not undergo DXA (*n* = 270) were excluded. We also excluded patients aged < 19 or > 90 years (*n* = 23) and those with a history of osteoporosis (*n* = 10). A total of 1,636 eligible participants were then classified as having TSCI (*n* = 1,159) or NTSCI (*n* = 475), excluding 2 individuals with an unknown aetiology (Fig. 1). Data collected included demographic factors (age at injury, sex, alcohol use, smoking history, and body mass index [BMI]) and clinical factors (time since injury, injury aetiology, neurological level of injury [NLI], American Spinal Cord Injury Association Impairment Scale [AIS], and Korean version of the Spinal Cord Independence Measure-III [K-SCIM-III]). The appropriate Institutional Review Board approved this study.

### Classification of non-traumatic and traumatic causes

The International SCI Data Sets standardize the collections and reporting of information on individuals with SCI (15). The aetiology of SCI can be categorized into traumatic and non-traumatic causes based on the International SCI Data Sets. NTSCI causes, as classified by the International SCI Non-Traumatic Data Set, include congenital conditions, non-traumatic degenerative aetiologies, tumours, vascular aetiologies, infections, and other non-traumatic spinal cord dysfunctions (16). TSCIs are classified as sports and leisure, assault, transport, fall, birth injury, or other traumatic causes based on the International Classification of External Causes of Injuries (17).

### DXA Measurements

Patients underwent DXA during a routine clinical evaluation at the rehabilitation centre. BMD was measured in the lumbar spine (L1‒L4), femoral neck, and total hip. Due to the policy of the National Health Insurance Services, insurance coverage for bone density tests is provided only in these 3 areas according to the WHO criteria (18). Bone sites with spinal implants or hip replacements were excluded. Two different DXA machines were used at the centre for our study, 1 before January 20, 2019, and 1 after that. Therefore, we used standardization formulas to convert data for consistency (19).

In 2013, the International Society for Clinical Densitometry calculated T-scores based on the National Health Nutritional Examination Survey III data, using young white females as reference values (20). According to the World Health Organization (WHO), the lowest diagnostic value of the measured lumbar and femur T-scores is used and categorized as normal (T-score –1.0), osteopenia (T-score < 1.0 and > –2.5), and osteoporosis (T-score ≤ –2.5) for subjects aged 50 years or older (18). For subjects under the age of 50 years, the Z-score was used to diagnose as normal (Z-score > –2) or below the expected range for age (Z-score ≤ –2) (21).

### Statistical analysis

All statistical analyses were performed using SPSS version 27.0 (IBM Corp, Armonk, NY, USA). Participant characteristics are presented as means and standard deviations (SD) for continuous variables and numerical values (n) and percentages (%) for categorical variables. Differences in characteristics according to age category between the NTSCI and TSCI groups and WHO diagnostic categories were assessed using the independent *t*-test and χ^2^ test. In addition, a two-sample *t*-test and χ^2^ test were used to compare the normal and osteoporosis groups based on the T-scores of the 2 groups. The effects of demographic and independent SCI variables on osteoporosis (in those over 50 years) and below the expected range for age (in those under 50 years) in the TSCI and NTSCI groups were estimated using hierarchical multiple regression analysis. We first developed Model 1, which included age, sex, and BMI, to explain the demographic variability of osteoporosis in each group. We then developed Model 2 with SCI variables (time since injury, NLI, AIS, and K-SCIM-III). The suitability of the regression model was confirmed by *R*^2^ (the coefficient of determination, which explains the variation in the dependent variable accounted for by the independent variable) and *p*-values*.* The Hosmer and Lemeshow test indicates a good-fitting model when its *p*-value is greater than 0.05. Statistical significance was set at *p <* 0.05.

## RESULTS

### Demographics and clinical characteristics

The demographic and clinical characteristics of the participants are presented in Table I. The cause of injury was non-traumatic in 475 individuals and traumatic in 1,159 individuals. Significant differences were found between the two groups in terms of age, sex, BMI, pre-injury alcohol use, smoking history, time since injury, NLI and K-SCIM-III scores based on *t*-test and χ^2^ test results (all *p <* 0.001).

**Table I:** Baseline demographics and clinical characteristics of participants (*n* = 1,634)

Category	NTSCI (*n* = 475) Mean (SD) or *n* (%)	TSCI (*n* = 1,159) Mean (SD) or *n* (%)	*p*-value
Age (years)	54.81 (15.7)	47.93 (15.2)	**< 0.001**
20–29	38 (8.0)	165 (14)	**< 0.001**
30–39	54 (11)	201 (17)	
40–49	67 (14)	244 (21)	
50–59	115 (24)	273 (23)	
60–69	110 (23)	185 (16)	
70–79	72 (15)	69 (6.0)	
80–89	19 (4.0)	22 (1.9)	
Sex			**< 0.001**
Male	270 (57)	922 (79)	
Female	205 (43)	237 (20)	
BMI (kg/m^2^)	23.38 (3.5)	22.45 (3.0)	**< 0.001**
Pre-injury alcohol use			**< 0.001**
None	424 (89)	916 (80)	
Yes	51 (11)	236 (20)	
Smoking history			**< 0.001**
None	363 (77)	740 (64)	
Yes	109 (23)	409 (36)	
Cause of TSCI			-
Transport		491 (42)	
Fall		531 (45)	
Assault		6 (0.5)	
Sports and leisure		37 (3.2)	
Other		94 (8.1)	
Cause of NTSCI			
Vertebral column degenerative disorder	113 (23)		
Neoplastic disorder	90 (18)		
Inflammatory and autoimmune disease	70 (14)		
Infection	48 (10)		
Vascular disorder	36 (7.6)		
Others	118 (24)		
Time since injury (months)			
< 12	3.69 (2.9)	4.13 (3.0)	**0.020**
≥ 12	57.11 (105.9)	51.41 (78.6)	0.553
NLI			**< 0.001**
Tetraplegia	155 (33)	736 (64)	
Paraplegia	318 (67)	419 (36)	
AIS			**< 0.001**
A	70 (14)	455 (39)	
B	40 (8.4)	192 (16)	
C	71 (14)	220 (19)	
D	289 (60)	286 (24)	
Motor score	69.05 (19.7)	44.50 (23.7)	**< 0.001**
UEMS	24.26 (16.0)	11.85 (15.5)	**< 0.001**
LEMS	44.79 (9.9)	32.65 (16.7)	**< 0.001**
K-SCIM-III	52.23 (23.0)	34.60 (23.1)	**< 0.001**
0–25	68 (14)	487 (42)	**< 0.001**
26–50	153 (32)	393 (33)	
51–75	165 (34)	208 (17)	
76–100	89 (18)	71 (6.1)	

AIS: Association Impairment Scale; BMI: body mass index; K-SCIM-III: Korean version of the spinal cord independence measure-III; LEMS: lower extremity motor score; NLI: neurological level of injury; NTSCI: non-traumatic spinal cord injury; SD: standard deviation; TSCI: traumatic spinal cord injury; UEMS: upper extremity motor score. Statistically significant *p*-values < 0.05 are shown in bold.

As shown in Fig. S1, there were differences in the rates of normal, osteopenia, and osteoporosis in the WHO diagnostic categories according to the cause of NTSCI.

### Differences in BMD and T-scores among bone sites according to WHO diagnostic categories

Table II indicates whether the BMD, T-score, and Z-score values for each region (the lumbar spine, femoral neck, and total hip) differed between the NTSCI and TSCI groups according to the WHO criteria. In groups over 50 years old, the lumbar spine BMD and T-score values were lower in the NTSCI group than in the TSCI group when osteoporosis was diagnosed (*p <* 0.05). However, there was no difference in BMD and T-score values between the 2 groups at other femoral sites in the over-50 age group and all diagnostic sites in the under-50 age group.

**Table II:** Results of DXA between the NTSCI and TSCI groups for diagnostic sites according to WHO diagnostic categories

≥ 50 years	Normal (*n* = 131)				Osteopenia (*n* = 411)			Osteoporosis (*n* = 323)			
	TSCI	NTSCI		*p*-value	TSCI	NTSCI	*p*-value	TSCI		NTSCI	*p*-value
Lumbar spine											
BMD (g/cm^2^)	1.16 (0.2)	1.10 (0.1)		0.065	0.93 (0.1)	0.92 (0.1)	0.469	0.80 (0.1)		0.75 (0.1)	**< 0.001**
T-score	1.03 (1.6)	0.52 (1.2)		0.065	–1.10 (0.9)	–1.17 (0.9)	0.469	–2.28 (1.3)		–2.77 (1.2)	**< 0.001**
Femur neck											
BMD (g/cm^2^)	0.89 (0.1)	0.86 (0.1)		0.180	0.66 (0.1)	0.66 (0.1)	0.887	0.54 (0.1)		0.52 (0.1)	0.219
T-score	0.23 (1.1)	–0.03 (0.8)		0.180	–1.66 (0.7)	–1.67 (0.6)	0.887	–2.70 (0.8)		–2.81 (0.8)	0.219
Total hip											
BMD (g/cm^2^)	1.01 (0.1)	1.00 (0.1)		0.683	0.85 (0.1)	0.84 (0.1)	0.233	0.67 (0.1)		0.65 (0.1)	0.249
T-score	0.61 (1.0)	0.53 (1.0)		0.683	–0.72 (0.9)	–0.83 (0.9)	0.233	–2.21 (1.1)		–2.34 (1.0)	0.249
< 50 years	Normal (*n* = 599)						Below the expected range of age (*n* = 170)				
	TSCI		NTSCI		*p*-value		TSCI		NTSCI		*p*-value
Lumbar spine											
BMD (g/cm^2^)	0.98 (0.1)		0.96 (0.1)		0.109		0.86 (0.1)		0.85 (0.1)		0.520
Z-score	–0.65 (0.9)		–0.80 (0.7)		0.108		–1.85 (1.0)		–2.18 (0.9)		0.063
Femur neck											
BMD (g/cm^2^)	0.72 (0.1)		0.73 (0.1)		0.227		0.59 (0.1)		0.60 (0.2)		0.789
Z-score	–0.41 (1.0)		–0.26 (0.9)		0.125		–1.58 (0.9)		–1.59 (1.2)		0.944
Total hip											
BMD (g/cm^2^)	0.86 (0.2)		0.88 (0.2)		0.176		0.67 (0.2)		0.72 (0.2)		0.105
Z-score	–0.15 (1.1)		–0.02 (1.1)		0.237		–1.69 (1.2)		–1.30 (1.5)		0.084

BMD: bone mineral density; DXA: dual-energy X-ray absorptiometry; NTSCI: non-traumatic spinal cord injury; TSCI: traumatic spinal cord injury; WHO: World Health Organization. Statistically significant *p*-values < 0.05 are shown in bold.

### Diagnostic bone sites for osteopenia and osteoporosis in the NTSCI and TSCI groups over 50 years old

**Fig. 2:**
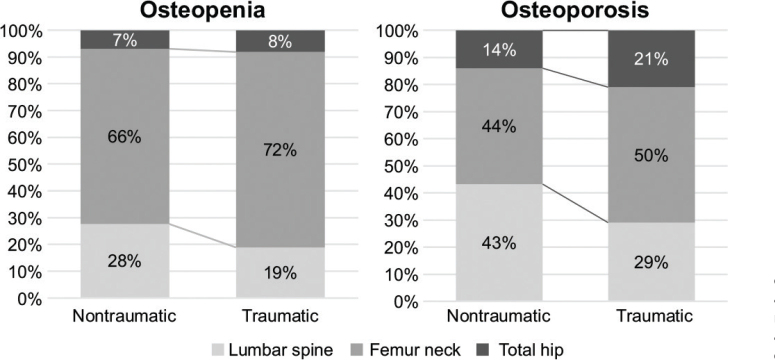
Lowest value among the diagnostic sites for osteopenia and osteoporosis between the non-traumatic spinal cord injury and traumatic spinal cord injury groups (over 50 years).

Fig. 2 shows the diagnostic bone sites for osteopenia and osteoporosis among the lumbar spine, femoral neck, and total hip sites. The lowest T-score value of the 3 measured areas becomes the diagnostic value. The diagnostic sites for osteopenia and osteoporosis were the femoral neck, followed by the lumbar spine and total hip in both groups, respectively. The lumbar spine had a higher proportion of diagnostic sites for osteopenia in the NTSCI group (28%) than in the TSCI group (19%). For osteoporosis, the lumbar spine had a higher proportion of diagnostic sites in the NTSCI group (43%) than in the TSCI group (29%).

### Differences between the normal and osteoporosis categories in the NTSCI and TSCI groups

The demographic and clinical characteristics of patients with osteoporosis compared with normal controls in the NTSCI and TSCI groups are given in Table III. Osteoporosis in the NTSCI group (≥ 50 years) was significantly associated with older age (*p =* 0.005), female sex (*p <* 0.001), and low BMI (*p =* 0.046). In the TSCI group (≥ 50 years), osteoporosis was significantly associated with female sex (*p <* 0.001), low BMI (*p =* 0.007), and tetraplegia (*p =* 0.032). An age below the expected range was significantly associated with a long duration of injury (*p <* 0.001) in the under-50 TSCI group.

**Table III:** Differences between the normal and osteoporosis categories in the NTSCI and TSCI groups

≥ 50 years T-score	NTSCI (*n* = 193)			TSCI (*n* = 261)		
	≥ –1.0 (*n* = 39)	≤ –2.5 (*n* = 154)	*p*-value	≥ –1.0 (*n* = 62)	≤ –2.5 (*n* = 169)	*p*-value
Gender						
Male	31 (80)	50 (33)	**< 0.001**	84 (91)	101 (60)	**< 0.001**
Female	8 (21)	104 (68)		8 (8.7)	68 (40)	
BMI (kg/m^2^)	24.22 (3.9)	23.04 (3.3)	**0.046**	23.28 (2.6)	22.37 (3.2)	**0.007**
Preinjury alcohol						
None	33 (85)	144 (94)	0.099	76 (83)	143 (86)	0.448
Yes	6 (15)	10 (6.5)		16 (17)	23 (14)	
Smoking history						
None	32 (82)	132 (87)	0.444	61 (66)	127 (77)	0.064
Yes	7 (18)	20 (13)		31 (34)	38 (23)	
Time since injury (months)						
< 12	35 (90)	120 (78)	0.097	70 (76)	110 (65)	0.067
≥ 12	4 (10)	34 (22)		22 (24)	59 (35)	
NLI						
Tetraplegia	15 (39)	45 (29)	0.265	74 (80)	115 (68)	**0.032**
Paraplegia	24 (62)	109 (71)		18 (20)	54 (32)	
AIS						
Motor complete	7 (18)	26 (17)	0.928	41 (45)	66 (40)	0.430
Motor incomplete	32 (82)	124 (83)		51 (55)	101 (61)	
K-SCIM-III	53.77 (22.6)	48.21 (21.5)	0.155	32.77 (22.4)	32.52 (24.2)	0.935
< 50 years Z-score	NTSCI (*n* = 159)			TSCI (*n* = 610)		
	> –2.0 (*n* = 114)	≤ –2.0 (*n* = 45)	*p*-value	> 2.0 (*n* = 485)	≤ –2.0 (*n* = 125)	*p*-value
Gender						
Male	73 (64)	30 (67)	0.754	385 (79)	92 (74)	0.163
Female	41 (36)	15 (33)		100 (21)	33 (26)	
BMI (kg/m^2^)	22.65 (3.5)	24.06 (5.2)	0.097	22.20 (3.0)	22.61 (3.8)	0.274
Preinjury alcohol						
None	101 (89)	144 (91)	0.644	377 (78)	104 (83)	0.235
Yes	13 (11)	10 (8.9)		104 (22)	21 (17)	
Smoking history						
None	79 (69)	35 (78)	0.285	290 (60)	74 (60)	0.881
Yes	35 (31)	10 (22)		190 (40)	50 (40)	
Time since injury (months)						
< 12	87 (76)	28 (62)	0.074	343 (71)	54 (43)	**< 0.001**
≥ 12	27 (24)	17 (38)		142 (29)	71 (57)	
NLI						
Tetraplegia	40 (35)	10 (22)	0.116	257 (53)	64 (51)	0.705
Paraplegia	74 (65)	35 (78)		227 (47)	61 (49)	
AIS						
Motor complete	39 (34)	12 (27)	0.403	341 (71)	85 (68)	0.528
Motor incomplete	75 (66)	32 (73)		140 (29)	40 (32)	
K-SCIM-III	53.77 (23.7)	55.24 (28.2)	0.689	36.09 (22.8)	35.01 (22.3)	0.634

^*^Motor complete injury (AIS A, B);^†^Motor incomplete injury (AIS C, D).

AIS: Association Impairment Scale; BMI: body mass index; K-SCIM-III: Korean version of the spinal cord independence measure-III; NLI: neurological level of injury; NTSCI: non-traumatic spinal cord injury; TSCI: traumatic spinal cord injury. Statistically significant *p*-values < 0.05 are shown in bold.

### Factors affecting osteoporosis in the NTSCI and TSCI groups among demographic and SCI variables

A hierarchical multiple regression analysis was performed to identify the factors affecting osteoporosis in the NTSCI and TSCI groups (Table IV). Model 1 used demographic factors as control variables to assess the effect on osteoporosis (≥ 50 years) and below the expected range for age ( < 50 years). Model 2 used SCI-related factors to investigate whether these factors affect osteoporosis (≥ 50 years) and below the expected range for age ( < 50 years) even after controlling for demographic factors. The overall multiple regression analysis in Model 1 was statistically significant (Hosmer–Lemeshow *=* 6.60, *p =* 0.580, *R*^2^ = 0.30), accounting for 30% of the demographic variables for non-traumatic osteoporosis. In addition, the TSCI group showed statistically significant differences (Hosmer–Lemeshow *=* 5.16, *p =* 0.740, *R*^2^ = 0.22), accounting for 22% of the variance in the development of osteoporosis. Female sex (*p <* 0.001, *p <* 0.001) and low BMI (*p =* 0.020, *p* = 0.017) were significant predictors of osteoporosis in NTSCI and TSCI groups, respectively. In addition, older age (*p =* 0.013) was a significant predictors of osteoporosis in the NTSCI group. The overall regression analysis for Model 2 was statistically significant (Hosmer–Lemeshow *=* 4.01, *p =* 0.856, *R*^2^ = 0.36), accounting for 36% of the variance in non-traumatic osteoporosis after adjusting for demographic factors. In addition, osteoporosis was statistically significant in the TSCI group (Hosmer–Lemeshow *=* 3.26, *p =* 0.917, *R*^2^ = 0.27), accounting for 27% of the variance, while time since injury accounted for an additional 5% of the variance after adjusting for demographic variables. A period of over 12 months after injury (*p =* 0.054, *p =* 0.005) was significantly associated with osteoporosis in both the NTSCI and TSCI groups. NLI, AIS, and K-SCIM-III scores did not significantly influence the results of the hierarchical multiple regression models.

**Table IV:** Hierarchical multiple regression analysis for osteoporosis in the NTSCI and TSCI groups

≥ 50 years	NTSCI				TSCI			
	Model 1		Model 2		Model 1		Model 2	
	OR	*p*-value	OR	*p*-value	OR	*p*-value	OR	*p*-value
(Constant)	1.11	0.958	0.93	0.976	6.18	0.246	6.86	0.235
Demographic variable								
Age (years)	1.06	**0.013**	1.08	**0.004**	1.03	0.158	1.03	0.169
Sex								
Male	(ref)		(ref)		(ref)		(ref)	
Female	8.79	**< 0.001**	9.90	**< 0.001**	8.15	**< 0.001**	8.24	**< 0.001**
BMI (kg/m^2^)	0.86	**0.020**	0.85	**0.017**	0.87	**0.004**	0.84	**0.001**
SCI variable								
Time since injury (months)								
< 12			(ref)				(ref)	
≥ 12			3.54	**0.054**			2.58	**0.005**
NLI								
Tetraplegia			(ref)				(ref)	
Paraplegia			1.37	0.524			1.93	0.100
AIS								
Motor complete			(ref)				(ref)	
Motor incomplete			0.60	0.407			1.59	0.197
K-SCIM-III			0.99	0.176			0.99	0.381
Nagelkerke *R*^2^	0.30		0.36		0.22		0.27	
Hosmer–Lemeshow	6.60 (0.580)		4.01 (0.856)		5.16 (0.740)		3.26 (0.917)	

< 50 years	NTSCI				TSCI			
	Model 1		Model 2		Model 1		Model 2	
	OR	*p*-value	OR	*p*-value	OR	*p*-value	OR	*p*-value

(Constant)	0.05	**0.038**	0.02	**0.008**	0.16	**0.021**	0.09	**0.005**
Demographic variable								
Age (years)	0.99	0.930	1.00	0.864	0.98	0.079	0.98	0.088
Sex								
Male	(ref)		(ref)		(ref)		(ref)	
Female	1.13	0.750	1.28	0.544	1.49	0.094	1.52	0.091
BMI (kg/m^2^)	1.09	0.061	1.10	0.059	1.05	0.100	1.05	0.129
SCI variable								
Time since injury (months)								
< 12			(ref)				(ref)	
≥ 12			2.44	**0.029**			3.54	**< 0.001**
NLI								
Tetraplegia			(ref)				(ref)	
Paraplegia			1.70	0.237			1.37	0.201
AIS								
Motor complete			(ref)				(ref)	
Motor incomplete			1.56	0.347			1.70	**0.036**
K-SCIM-III			0.99	0.605			0.99	0.162
Nagelkerke *R*^2^	0.03		0.09		0.02		0.27	
Hosmer–Lemeshow	4.06 (0.852)		8.68 (0.370)		5.81 (0.668)		5.43 (0.711)	

^*^Motor complete injury (AIS A, B); †Motor incomplete injury (AIS C, D).

AIS: Association Impairment Scale; BMI: body mass index; K-SCIM-III: Korean version of the spinal cord independence measure-III; NLI: neurological level of injury; NTSCI: non-traumatic spinal cord injury; OR: odds ratio; SCI: spinal cord injury; TSCI: traumatic spinal cord injury. Statistically significant *p*-values < 0.05 are shown in bold.

The overall multiple regression analysis in Model 1 was statistically significant (Hosmer–Lemeshow *=* 4.06, *p =* 0.852, *R*^2^ = 0.03), accounting for only 3% of the variance in demographic variables related to non-traumatic cases below the expected age range (< 50 years). In addition, the NTSCI group showed statistically significant differences (Hosmer–Lemeshow *=* 5.81, *p =* 0.668, *R*^2^ = 0.02), accounting for 2% of the variance in development below the expected range for age. None of the demographic or SCI variables were significant predictors of scores below the expected range for age in the NTSCI and TSCI groups ( < 50 years), respectively. The overall regression analysis for Model 2 was statistically significant (Hosmer–Lemeshow *=* 8.68, *p =* 0.37, *R*^2^ = 0.09), accounting for 9% of the variance in non-traumatic cases below the expected range for age ( < 50 years), and time since injury accounted for an additional 6% of the variance after adjusting for demographic factors. In addition, osteoporosis was statistically significant in the TSCI group (Hosmer–Lemeshow *=* 5.81, *p =* 0.668, *R*^2^ = 0.27), accounting for 27% of the variance, and time since injury and motor completeness accounted for an additional 24% of the variance after adjusting for demographic variables. A period of over 12 months after injury (*p =* 0.029, *p <* 0.001) was significantly associated with below the expected range for age in the NTSCI and TSCI groups. In addition, complete motor injury (*p =* 0.036) was a significant predictor of outcomes below the expected range for age in TSCI group. NLI and K-SCIM-III scores did not significantly influence the results of the hierarchical multiple regression models.

## DISCUSSION

The major finding of the current study is that the BMD of patients with NTSCI showed different DXA results compared with those with TSCI. In patients over 50 years old with NTSCI, the lumbar spine showed lower BMD than in those with TSCI and had a higher proportion of diagnostic sites for osteopenia and osteoporosis. This suggests that the lumbar spine is a major site of bone density reduction in NTSCI patients. We also found a greater risk of osteoporosis development in NTSCI and TSCI patients (≥ 50 years) with similar demographic characteristics – such as older age, female sex, and low BMI – compared with SCI-related factors, except for the time since injury.

Notably, most studies on BMD describe patients with TSCI. However, due to its increasing global incidence and prevalence, it is necessary to investigate NTSCI (14, 22). Developed countries are more likely to have a higher number of cases of degenerative diseases and tumours that cause NTSCI. Developing countries tend to have higher rates of infections, such as tuberculosis and human immunodeficiency virus (23). Compared with patients with TSCI, those with NTSCI show significant differences in age, sex, injury level, and severity (14, 22). We also found that individuals with NTSCI were older, more likely to be female, had a higher BMI, less pre-injury alcohol use, less smoking history, a shorter duration of injury, paraplegia, incomplete motor injury (AIS C and D), higher motor scores, and higher K-SCIM-III scores than those with TSCI. This implies that NTSCI and TSCI represent distinct demographic and clinical groups, suggesting that diagnostic sites for bone loss and therapeutic approaches should also be differentiated accordingly.

The most significant differences in BMD and T-score between the TSCI and NTSCI groups (over 50 years) were observed in the lumbar spine. Multiple studies have recommended DXA scans of the total hip, proximal tibia, and femur for BMD in adults with SCI but not of the lumbar spine (23–25). The BMD values at the lumbar spine in patients with TSCI were normal or minimally decreased, which is related to wheelchair use and allows the truncal weight to be loaded onto the lumbar spine (26). However, in our study, the BMD and T-score values of the lumbar spine in patients with NTSCI were significantly lower than those of patients with TSCI among the normal, osteopenia, and osteoporosis groups. In addition, the lowest values at the diagnostic sites for osteopenia and osteoporosis showed different rates between the NTSCI and TSCI groups. Compared with TSCI, the lumbar spine had a higher proportion of diagnostic sites for both osteopenia and osteoporosis in the NTSCI group. The NTSCI group had a higher proportion of paraplegia, which has a protective effect against the development of osteoporosis due to continued weightbearing on the lumbar area during wheelchair use (27). However, in this study, the lumbar BMD and T-score values of NTSCI patients were significantly lower than those of TSCI patients across all groups: normal, osteopenia, and osteoporosis. Additionally, the NTSCI group showed a higher proportion of diagnostic sites for osteopenia and osteoporosis in the lumbar spine.

This suggests that lumbar bone loss in the NTSCI group, unlike in the TSCI group, may have occurred for the following reasons: non-traumatic spinal cord injuries are primarily caused by irreversible functional losses such as infections, tumours, and degenerative conditions, leading to long-term and progressive damage (28, 29). Such chronic conditions may gradually induce bone loss, and the lumbar spine, with its higher proportion of trabecular bone, which is more susceptible to bone resorption, could be particularly sensitive to these changes (30, 31). Moreover, the demographic characteristic of NTSCI patients being older than TSCI patients is also expected to have influenced the lumbar spine, which is significantly affected by age-related metabolic decline (14, 32).

Therefore, it is plausible that lumbar bone loss is more pronounced in NTSCI patients than femoral bone loss, which predominantly involves cortical bone. These findings highlight the importance of monitoring lumbar BMD in NTSCI patients over 50 years of age and emphasize the need for differentiated, tailored management strategies between the two groups.

Furthermore, several studies have investigated the association between age, sex, race, ambulatory status, time since injury, SCI severity, and the development of osteoporosis. However, clinical findings regarding the relationship between osteoporosis and its risk factors are inconsistent and controversial. The association between age and BMD area using DXA in patients with SCI has yielded mixed results (33). These studies have found no relationship, inverse relationship, or age-related decrease in BMD (8, 34, 35). Other risk factors have shown inconsistent associations with osteoporosis. Identifying the high-risk factors for osteoporosis is difficult because SCI is a significant risk factor (33).

Both demographic and SCI-related factors are associated with the development of osteoporosis and can also affect the interaction between factors contributing to osteoporosis. In the general population, demographic factors strongly influence osteoporosis. We used hierarchical multiple analyses to identify demographic factors associated with osteoporosis and demonstrated the effect of SCI on osteoporosis after adjusting for these factors. Among the demographic factors, older age, female sex, and low BMI were associated with the development of osteoporosis in patients with NTSCI (over 50 years). TSCI has similar osteoporotic risk factors, such as sex, BMI, and time since injury. Except for time since injury, older age, female sex, and low BMI are critical predictors of osteoporosis in patients at high risk of SCI, similar to those in the general healthy population.

This study had some limitations. First, the SCI patients included in this study were recruited from a single centre, and a larger sample size is needed to clearly distinguish the differences between the NTSCI and TSCI groups. Second, the retrospective cross-sectional study design limited the ability to control for variables influencing the interpretation of risk factors. Consequently, data potentially affecting osteoporosis development, such as diet, family history, disease (chronic kidney disease, hyperthyroidism, etc.), and medications (glucocorticoid therapy, teriparatide, etc.), were not collected. The patient screening test could not be performed in advance; therefore, degenerative changes in the spine – including osteophytes and aortic calcification, severe scoliosis, and vertebral fractures – that may affect bone density values could not be taken into account. Third, according to the official guidelines of the International Society for Clinical Densitometry (ISCD), DXA scans at the total hip, proximal tibia, and distal femur are recommended for BMD measurement in SCI patients (33). However, this study focused on osteoporosis diagnostic criteria used in clinical practice, including those for the general population, and measured BMD at the lumbar spine, femoral neck, and total hip (36).Therefore, further research focusing on the proximal tibia and distal femur, which are at higher risk for osteoporotic fractures in SCI patients, is needed.

In conclusion, our results showed that patients over 50 years old with NTSCI have different bone mineral density and osteoporotic diagnostic sites compared with those with TSCI. BMD measurement in patients over 50 years old with NTSCI requires the assessment of additional diagnostic areas, particularly the lumbar spine. Both the NTSCI and TSCI groups (aged ≥ 50 years) had significant risk factors for osteoporosis, namely older age, female sex, low BMI, and being more than 1 year post-SCI. Further prospective studies with larger samples are required to address the limitations of this study.

## Supplementary Material


