# Supplementary material for: DIFFERENT APPROACH TO BONE MINERAL DENSITY IN NON-TRAUMATIC SPINAL CORD INJURY: A COMPARISON WITH TRAUMATIC SPINAL CORD INJURY
Source: J Rehabil Med. 2026 Mar 17;58:44836. doi: 10.2340/jrm.v58.44836 (PMC13005228; doi:10.2340/jrm.v58.44836)
Supplement: Supplementary file 1 [file JRM-58-44836-s1.pdf]

Fig S1. BMD diagnostic categories by non-traumatic causes

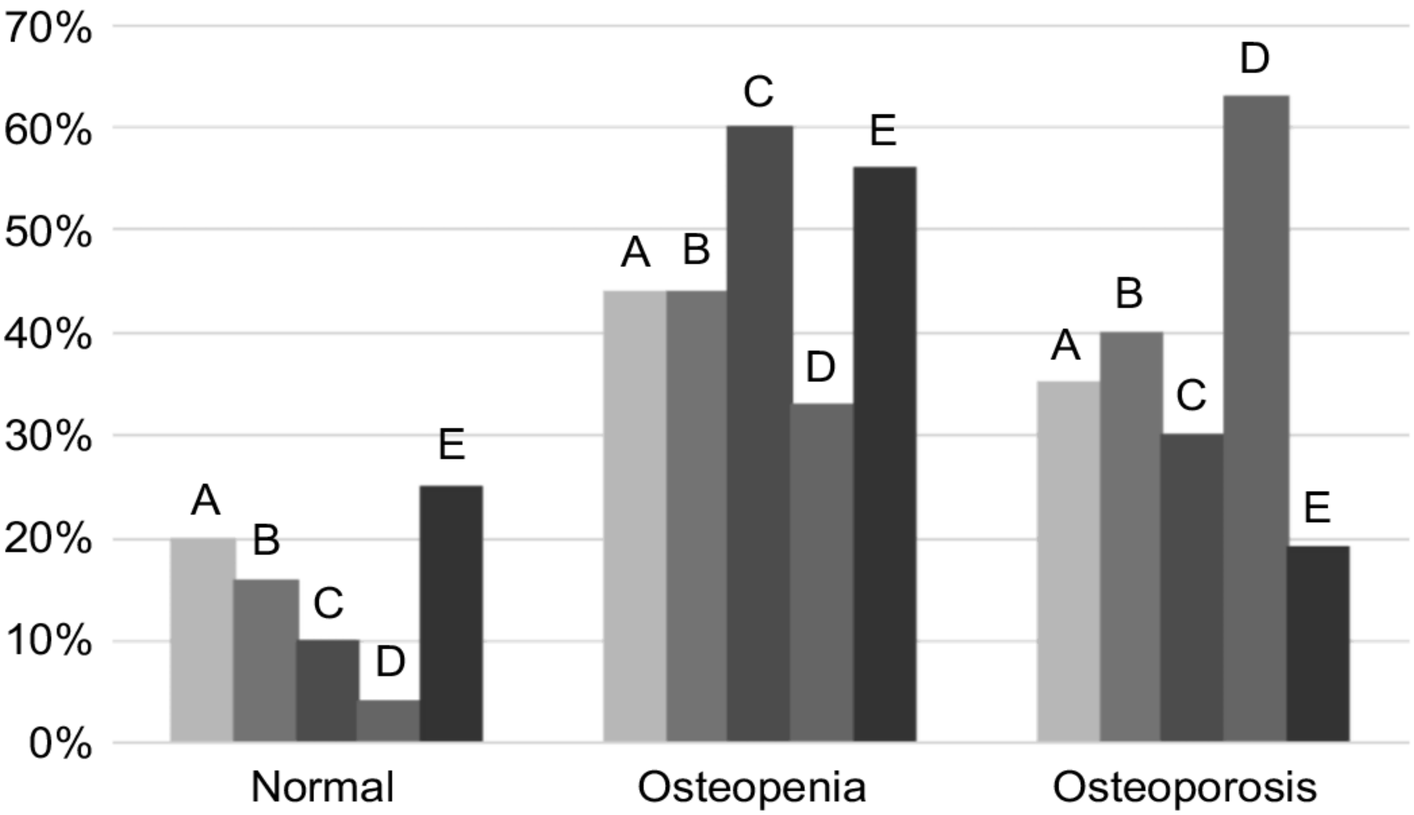

BMD, bone mineral density

A: Vertebral column degenerative disorder; B: Neoplastic disorder; C: Inflammatory and autoimmune disease; D: Infection; E: Vascular disorder
